# Supplementary material for: Delaying histone deacetylase response to injury accelerates conversion into repair Schwann cells and nerve regeneration
Source: Nat Commun. 2017 Jan 31;8:14272. doi: 10.1038/ncomms14272 (PMC5290322; doi:10.1038/ncomms14272)
Supplement: Supplementary Information — Supplementary Figures [file ncomms14272-s1.pdf]

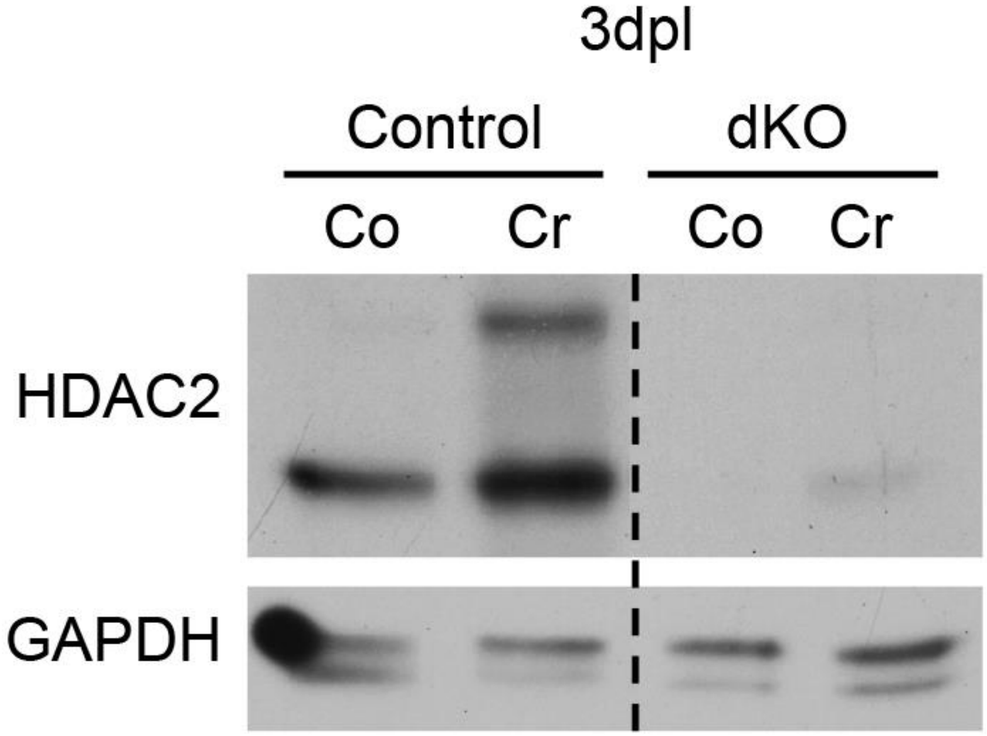

Supplementary Fig. 1 Both SUMOylated and non-SUMOylated forms of HDAC2 are lost in dKO. Western blot of HDAC2 and GAPDH (loading control) in crushed (Cr) and contralateral (Co) nerves of Control and dKO mice at 3dpl. Sample size: 5 animals per group. Representative photos are shown. Dashed line: samples were run on the same gel, but not on consecutive lanes.

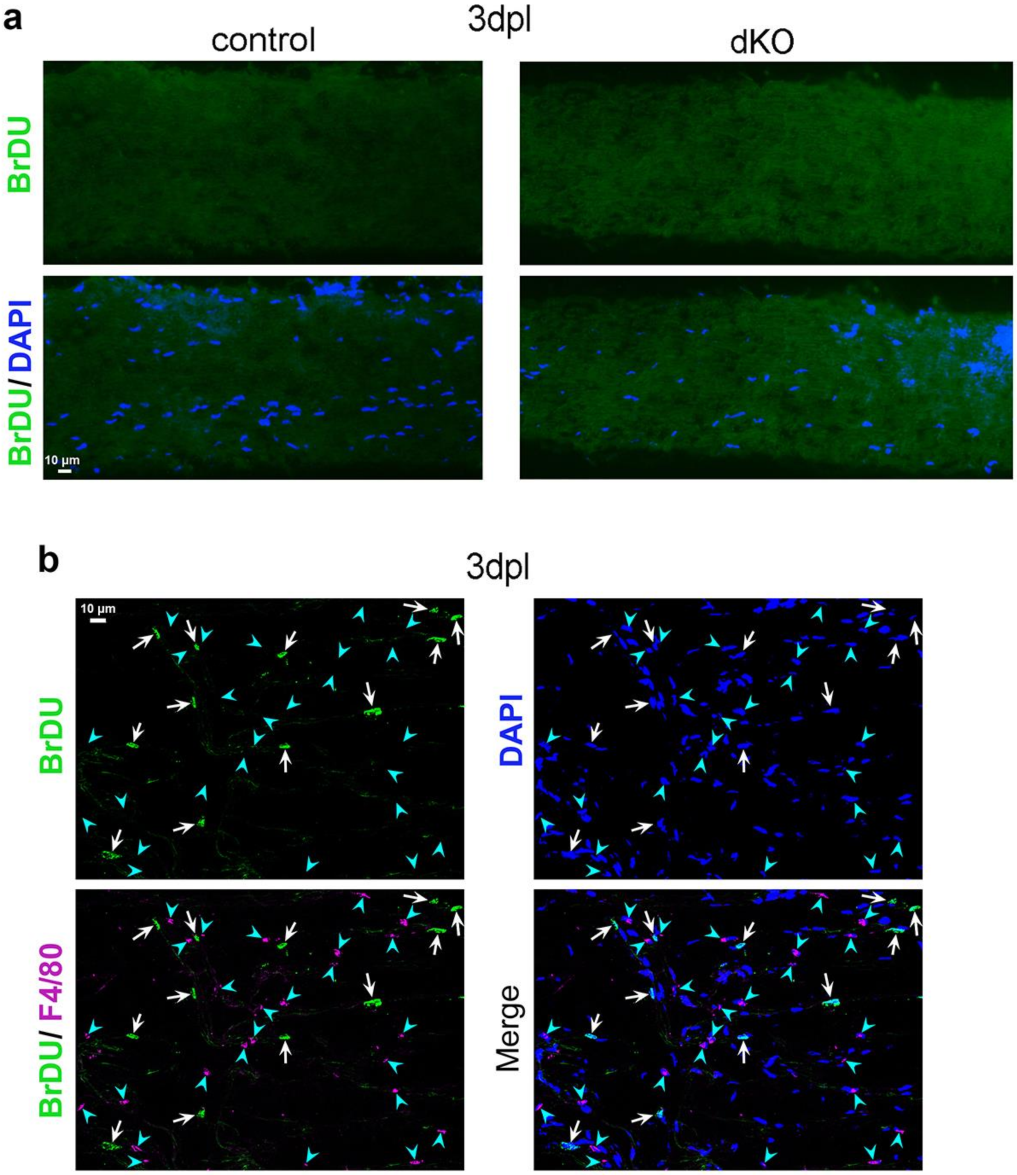

Supplementary Fig. 2 Proliferating cells at 3dpl in dKO crushed nerves are not macrophages and no increased proliferation was detected in contralateral nerves. (**a**) BrdU incorporation (green) and DAPI (blue) showing absence of BrDU-positive cells (proliferating cells) in control and dKO contralateral nerves at 3dpl. No BrDU-positive cell was detected either at 5dpl and 12dpl in contralateral nerves of control and dKO mice (not shown). (**b**) BrdU incorporation (green, white arrows) and co-labeling with the macrophage marker F4/80 (red, blue arrowheads) and DAPI (blue=nuclei) at 3dpl. Sample size: 3 animals per group. Representative photos are shown.

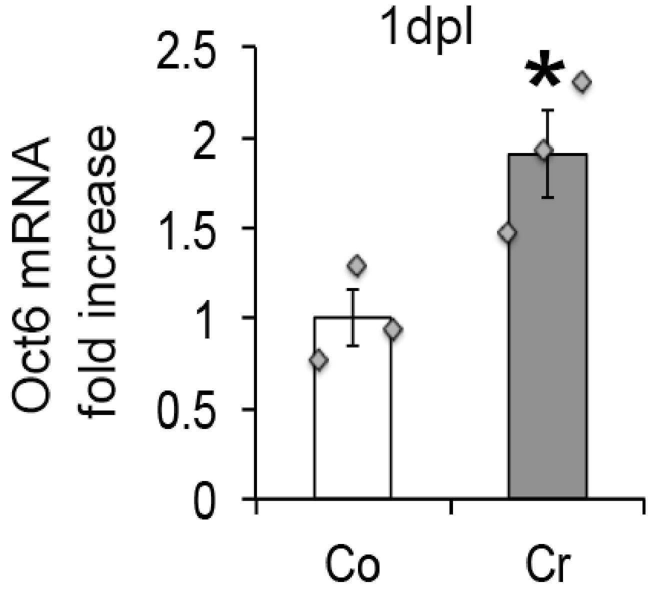

Supplementary Fig. 3 Oct6 is upregulated at the transcript level at 1dpl in sciatic nerves. *Oct6* mRNA fold increase normalized to GAPDH in crushed (Cr) compared to contralateral (Co) nerves of adult mice showing increased *Oct6* mRNA levels at 1dpl. Two-tailed unpaired Student's *t*-tests, *P* value=0.03. Sample size: 3 animals per group. Values=mean, error bars=s.e.m.

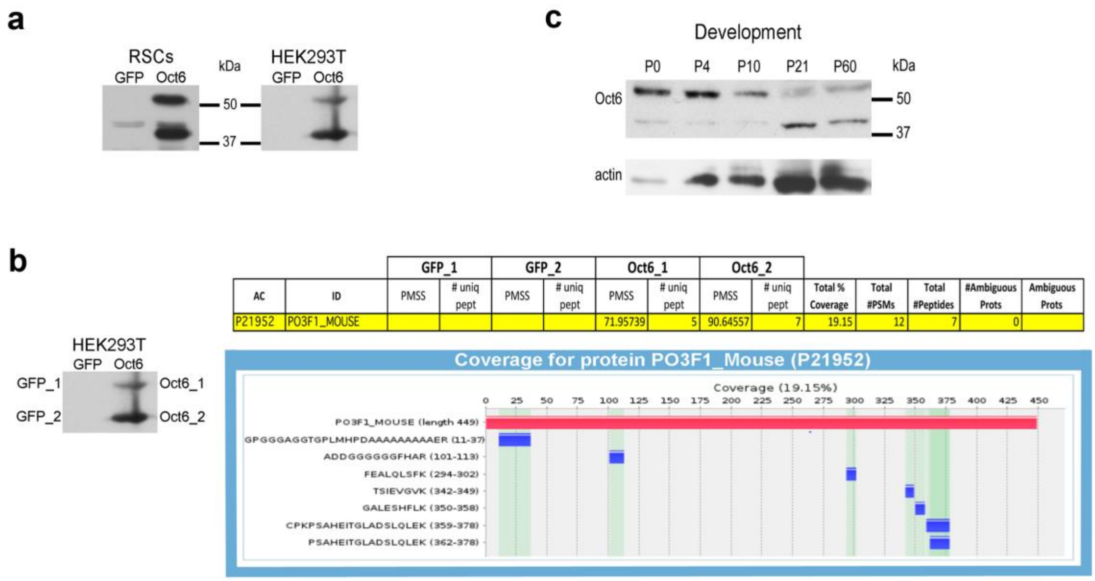

		GFP_1		GFP_2		Oct6_1		Oct6_2						
AC	ID	PMSS	# uniq pept	PMSS	# uniq pept	PMSS	# uniq pept	PMSS	# uniq pept	Total % Coverage	Total #PSMs	Total #Peptides	#Ambiguous Prots	Ambiguous Prots
P21952	PO3F1_MOUSE					71.95739	5	90.64557	7	19.15	12	7	0	

Supplementary Fig. 4 Validation of the two Oct6 isoforms regulated during development and after lesion and detected by the Oct6 antibody. (**a**) Western blot of Oct6 in primary rat Schwann cells (RSCs) and HEK293T cells after transfection with a GFP- or an Oct6-expressing constructs. (**b**) Mass spectrometry analysis of the two bands (Oct6_1 and Oct6_2) detected by Oct6 antibody in lysates of HEK293T cells 1 day after transfection with an Oct6-expressing construct. As controls, analysis of proteins running at the same sizes (GFP_1 and GFP_2) as Oct6_1 and Oct6_2 was carried out. For this control experiment, HEK293T cells, which do not endogenously express Oct6, were transfected with a GFP-expressing construct for 1 day. (**c**) Western blot of Oct6 and beta-actin (loading control) in lysates of developing mouse sciatic nerves at birth (P0) and at 4, 10, 21, and 60 postnatal days (P). Representative Western blot images of 3 series of mouse nerves (n=3 animals for each postnatal age).

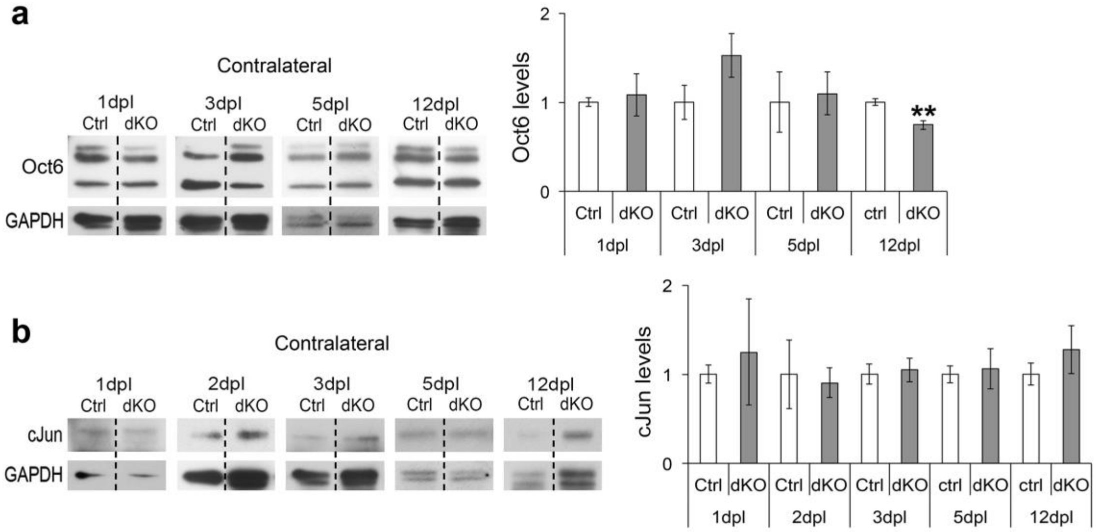

Supplementary Fig. 5 Oct6 and cJun levels remain mostly unaffected in dKO contralateral nerves. Western blots of Oct6 (**a**), cJun (**b**) and GAPDH (loading control) in contralateral nerves of control (Ctrl) and dKO mice at 1, 2, 3, 5 and 12dpl, and quantification of protein levels in dKO compared to Ctrl nerves. Dashed lines: samples run on the same gel, but not on consecutive lanes. Two-tailed (for Oct6 at 12dpl) or one-tailed unpaired Student's *t*-tests showing no significant difference between dKO and Ctrl, except for Oct6 at 12dpl. *P* value: **<0.01. Sample size: 5 animals per group at 1, 2 and 3dpl; 6 animals per group at 5 and 12dpl. Values=mean, error bars=s.e.m.

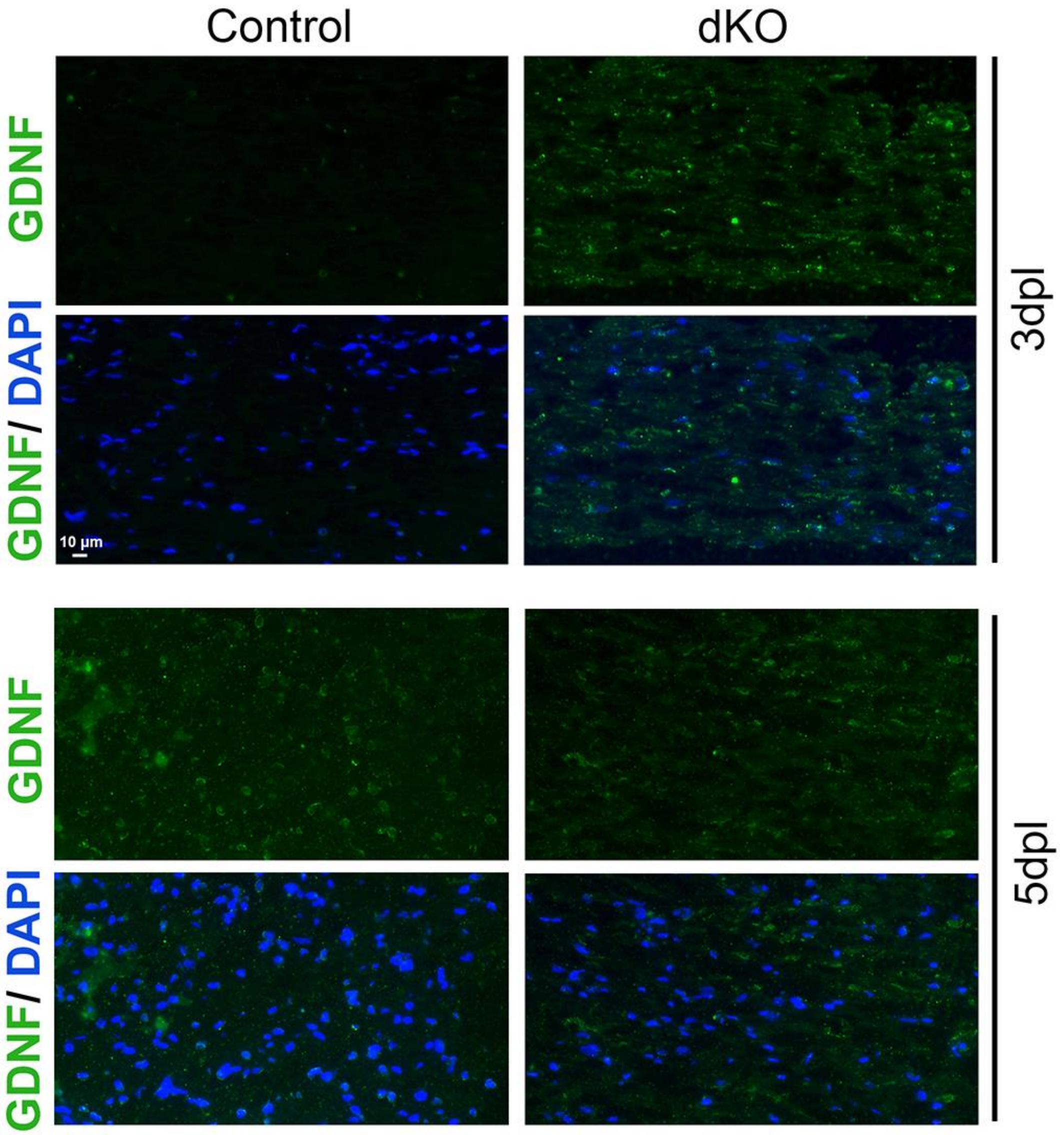

Supplementary Fig. 6 Earlier expression of GDNF in dKO compared to control nerves after lesion. Immunofluorescence of GDNF (green) and DAPI labeling (blue=nuclei) in dKO and control crushed nerves at 3 and 5dpl, showing GDNF expression in dKO nerves already at 3dpl, whereas GDNF expression was detectable later at 5dpl in control nerves. Sample size: 3 animals per group per time-point.

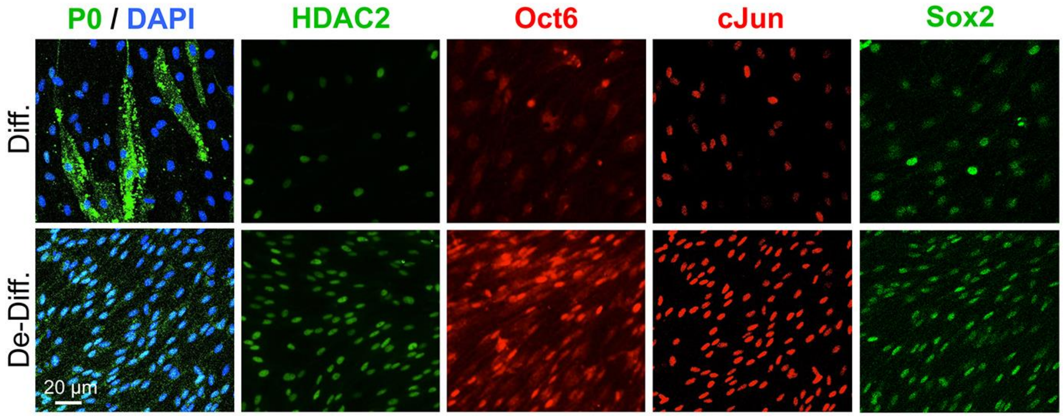

Supplementary Fig. 7 Validation of cell culture model of differentiation and dedifferentiation. Immunofluorescence of P0 (green) and DAPI labeling (blue=nuclei), or of HDAC2 (green), Oct6 (red), cJun (red) or Sox2 (green) in primary RSCs cultured to model differentiation (Diff.) or dedifferentiation (De-diff.), mimicking downregulation of P0 and upregulation of HDAC2, Oct6, cJun and Sox2 in dedifferentiation compared to differentiation culture conditions, such as after lesion in sciatic nerves of adult mice. Representative images of ≥4 experiments are shown.

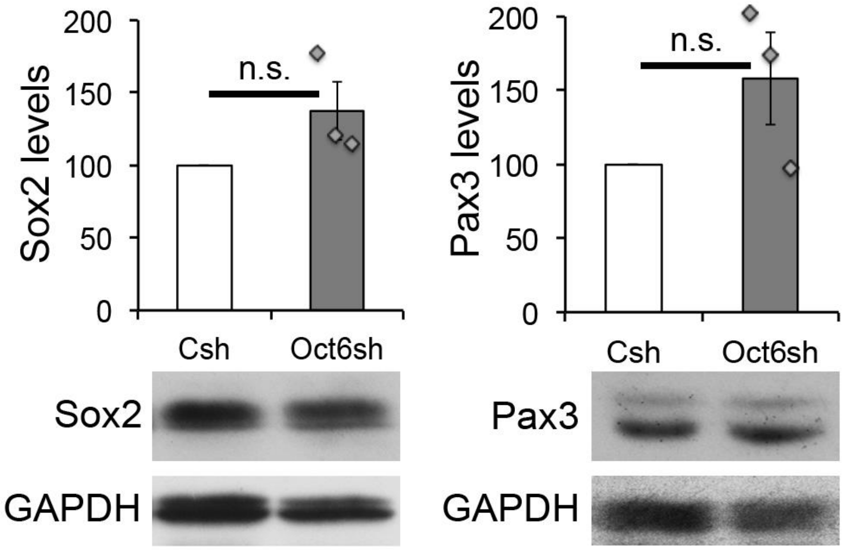

Supplementary Fig. 8 Sox2 and Pax3 levels are not significantly affected by Oct6 downregulation. Western blots of Sox2 and Pax3 in primary RSCs transduced with lentiviruses carrying either an Oct6 shRNA (Oct6sh) to downregulate Oct6 or a control shRNA (Csh). Graphs show the quantification of protein levels normalized to GAPDH in Oct6sh-transduced cells compared to Csh (=1). One-tailed paired Student's *t*-tests. Sample size: n=3. Values=mean, error bars=s.e.m.

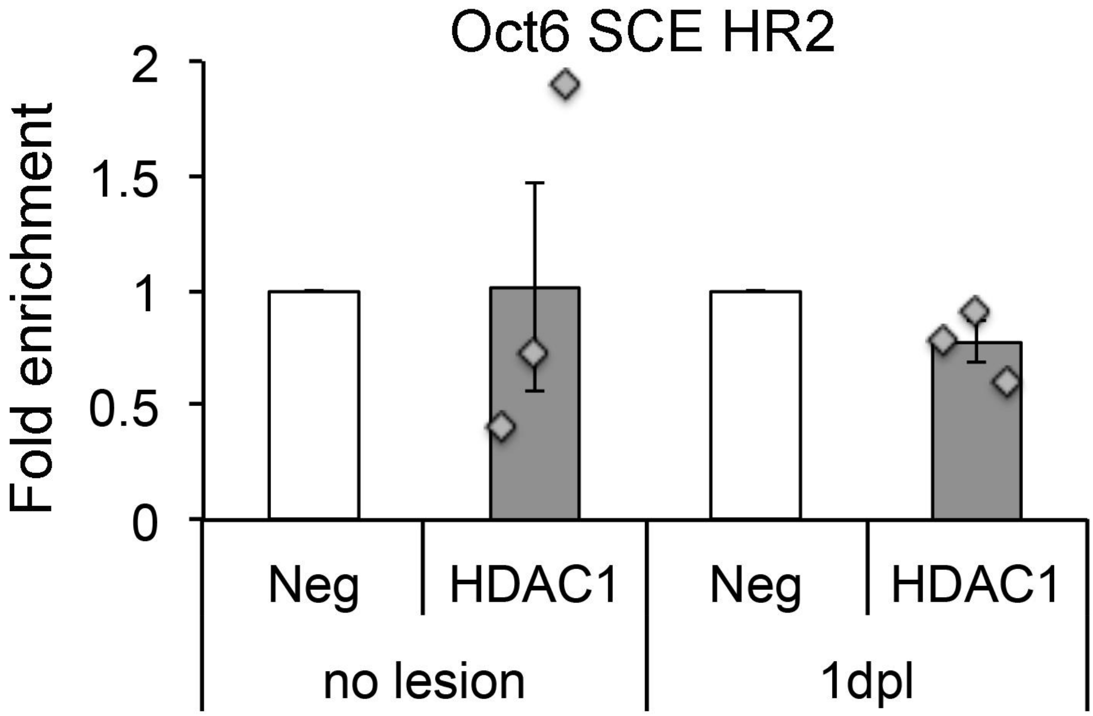

Supplementary Fig. 9 HDAC1 is not bound to the *Oct6* SCE HR2 at 1dpl. ChIP of HDAC1 and control GFP (Neg=1) in unlesioned (no lesion) nerves or at 1dpl on the *Oct6* SCE HR2 in sciatic nerves of control mice. One-tailed paired Student's *t*-test shows no significant difference between HDAC1 and Neg ChIP. Sample size: n=3. Values=mean, error bars=s.e.m.

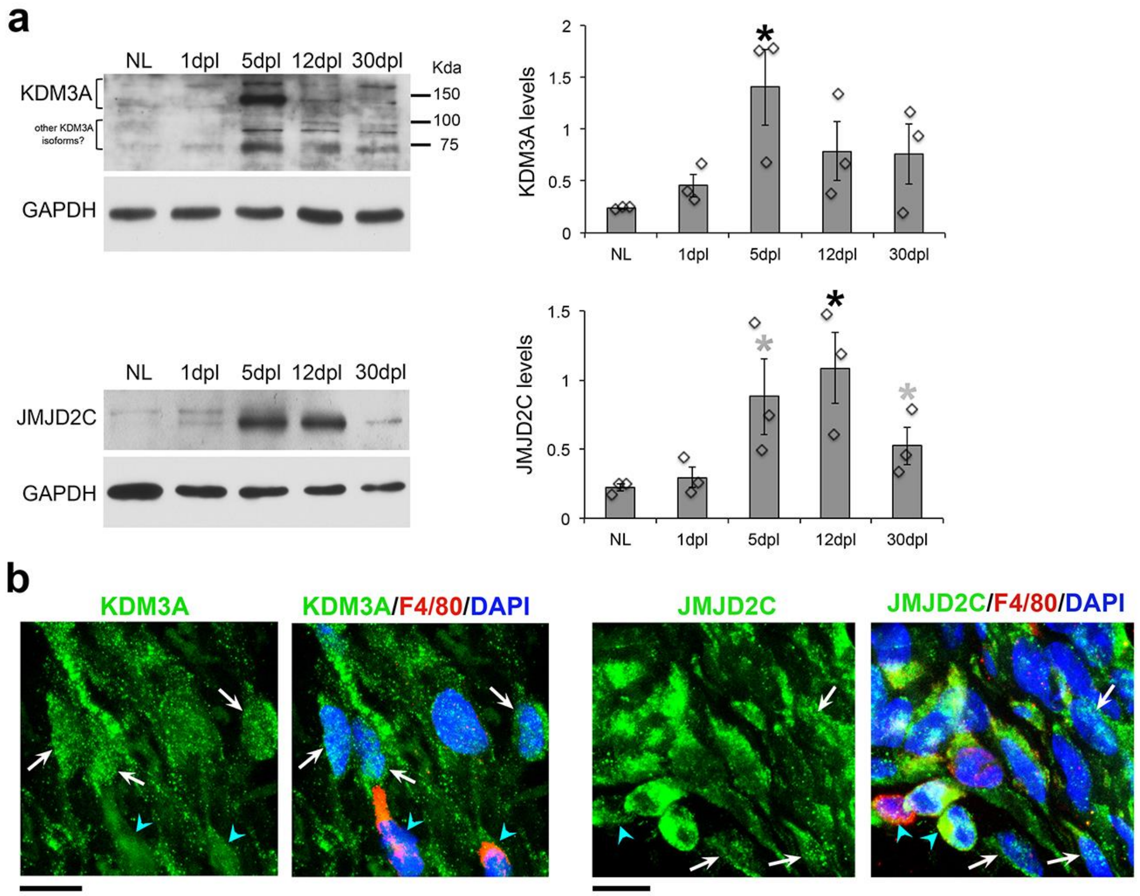

Supplementary Fig. 10 JMJD2C and KDM3A are expressed at low levels in adult sciatic nerves and regulated after lesion. (**a**) Western blots of KDM3A or JMJD2C and GAPDH (loading control) and quantification normalized to GAPDH in lysates of unlesioned (NL) adult mouse sciatic nerves or at 1, 5, 12, 30 days post lesion (dpl), showing for both KDM3A and JMJD2C low expression in unlesioned nerves and upregulation at 5dpl. Note that the KDM3A antibody recognizes several bands (some also detected with a second KDM3A antibody): two higher molecular weight bands at ~147 and 170 KDa (most described isoforms), and two lower molecular weight bands at ~95 and 75 KDa that could be other KDM3A isoforms or non-specific bands. Two-tailed (black asterisks) or one-tailed (grey asterisks) unpaired Student's *t*-tests showing significance compared to NL. *P* value: *<0.05. Sample size: 3 animals per group. Values=mean, error bars=s.e.m. (**b**) Z-series projections (confocal stacks) of KDM3A or JMJD2C (green) co-immunofluorescence with the macrophage marker F4/80 (red, overlay appears yellow) and DAPI labeling (blue=nuclei) in cryosections of adult mouse sciatic nerves at 5dpl. White arrows indicate SCs (F4/80-negative cells with elongated nuclei) and blue arrowheads indicate macrophages (F4/80-positive cells). Scale bars=10 µm. Note that KDM3A and JMJD2C are both nuclear and cytoplasmic and expressed in SCs and macrophages. Sample size: 3 animals. Representative photos are shown.

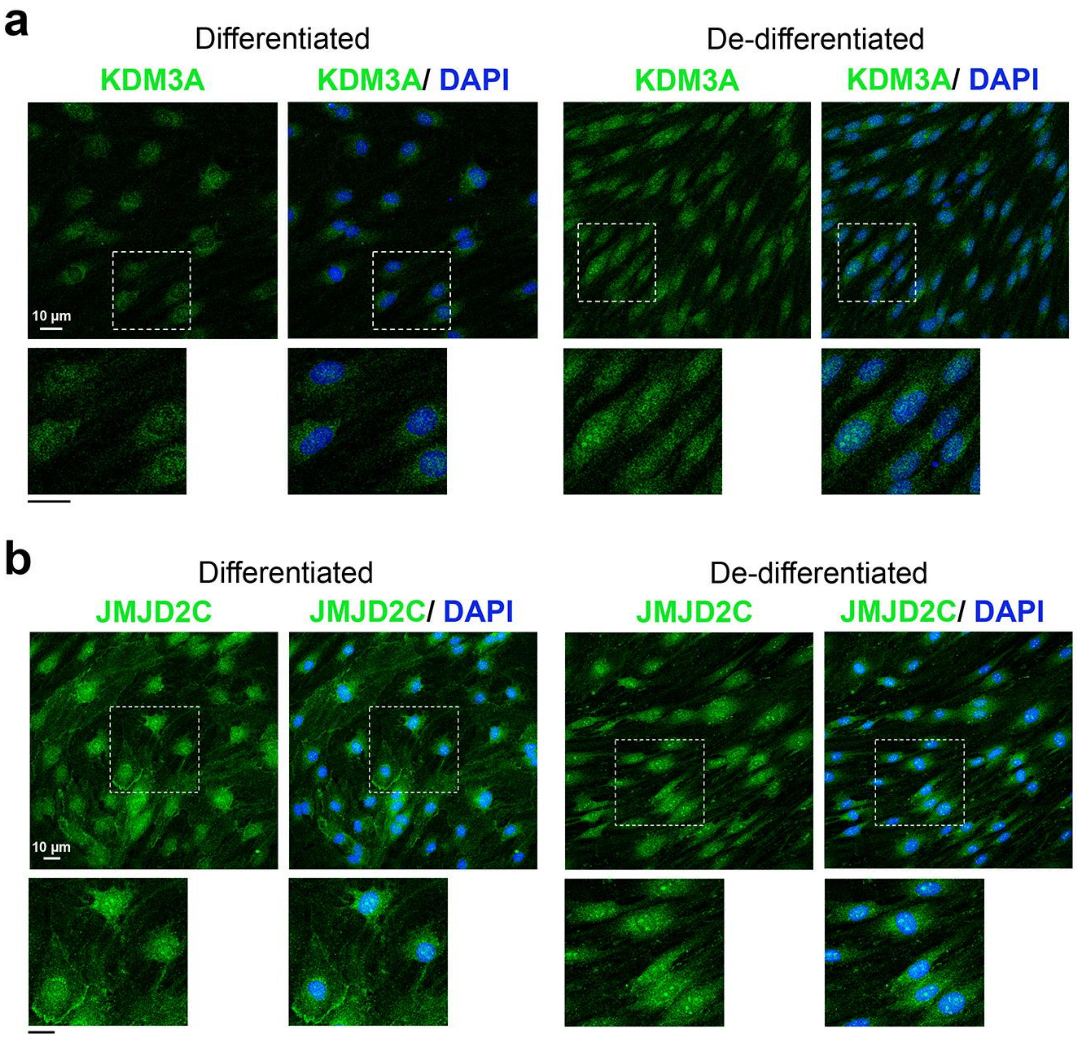

Supplementary Fig. 11 KDM3A and JMJD2C are expressed in differentiated and de-differentiated RSCs. Immunofluorescence of KDM3A (green, **a**) or JMJD2C (green, **b**) and DAPI labeling (nuclei) in primary RSCs cultured under differentiating and de-differentiating conditions. Lower photos of each panel are magnifications of the white boxes. Scale bars=10 µm. Note that KDM3A and JMJD2C are both nuclear and cytoplasmic. Sample size: (**a**,**b**) n=3.

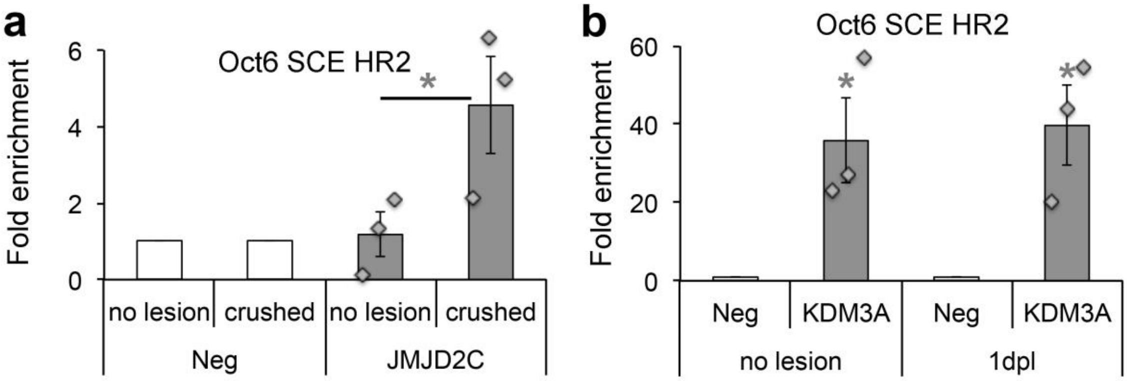

Supplementary Fig. 12 JMJD2C and KDM3A are enriched at the *Oct6* SCE HR2 at 1dpl. ChIP of (**a**) JMJD2C and Neg (=1, GFP) in unlesioned nerves or at 1dpl, and of (**b**) KDM3A and Neg (=1, Flag) in unlesioned nerves and at 1dpl on the *Oct6* SCE HR2 in sciatic nerves of control mice. One-tailed unpaired (**a**) or paired (**b**) Student's *t*-tests, *P* values: *<0.05. Asterisks indicate significance compared to no lesion (**a**) or to Neg (**b**). Sample size: n=3. Values=mean, error bars=s.e.m.

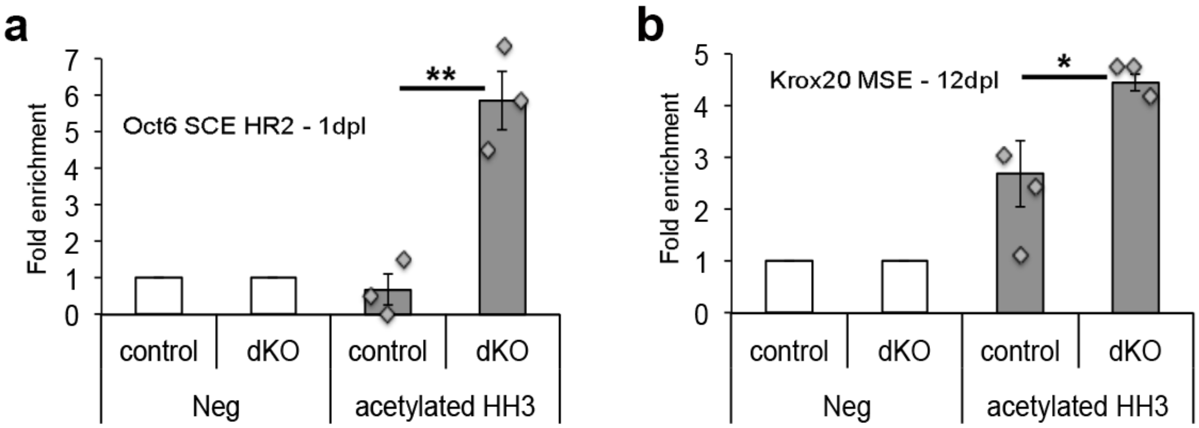

Supplementary Fig. 13 Consistent with HDAC known functions on histones, histone H3 acetylation is increased at the *Oct6* SCE HR2 and *Krox20* MSE in dKO nerves. ChIP of acetylated histone H3 on (**a**) the *Oct6* SCE HR2 at 1dpl and on (**b**) the *Krox20* MSE at 12dpl, in control and dKO nerves. Two-tailed unpaired Student's *t*-tests, *P* values: *<0.05, **<0.01. Sample size: (**a**,**b**) 3 mice per group control and dKO at each time-point 1dpl and 12dpl. Values=mean, error bars=s.e.m.

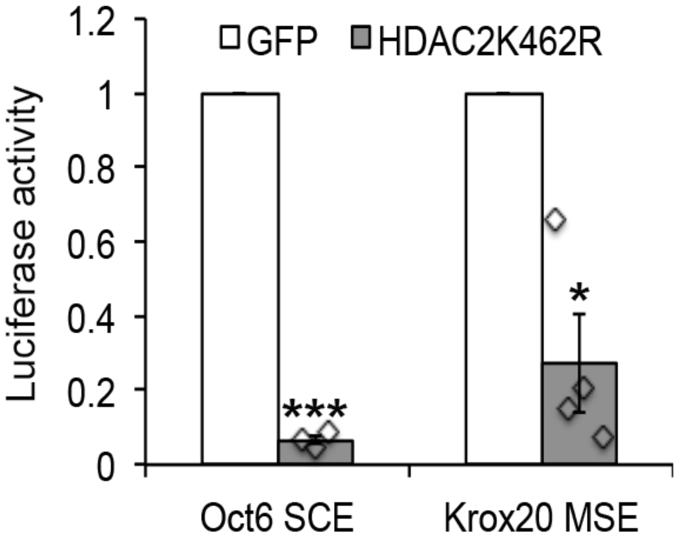

Supplementary Fig. 14 HDAC2 SUMOylation at K462 is critical for HDAC2-dependent activation of the *Oct6* SCE and the *Krox20* MSE. Relative luciferase activity of the full *Oct6* SCE, or the *Krox20* MSE, in primary rat Schwann cells transfected with GFP (=1) or HDAC2K462R mutant expression constructs and cultured under dedifferentiating **(***Oct6* SCE) or redifferentiating **(***Krox20* MSE) conditions for 1 day. Two-tailed paired Student's *t*-tests, *P* values: *<0.05, ***<0.001. Sample size: n=3. Values=mean, error bars=s.e.m.

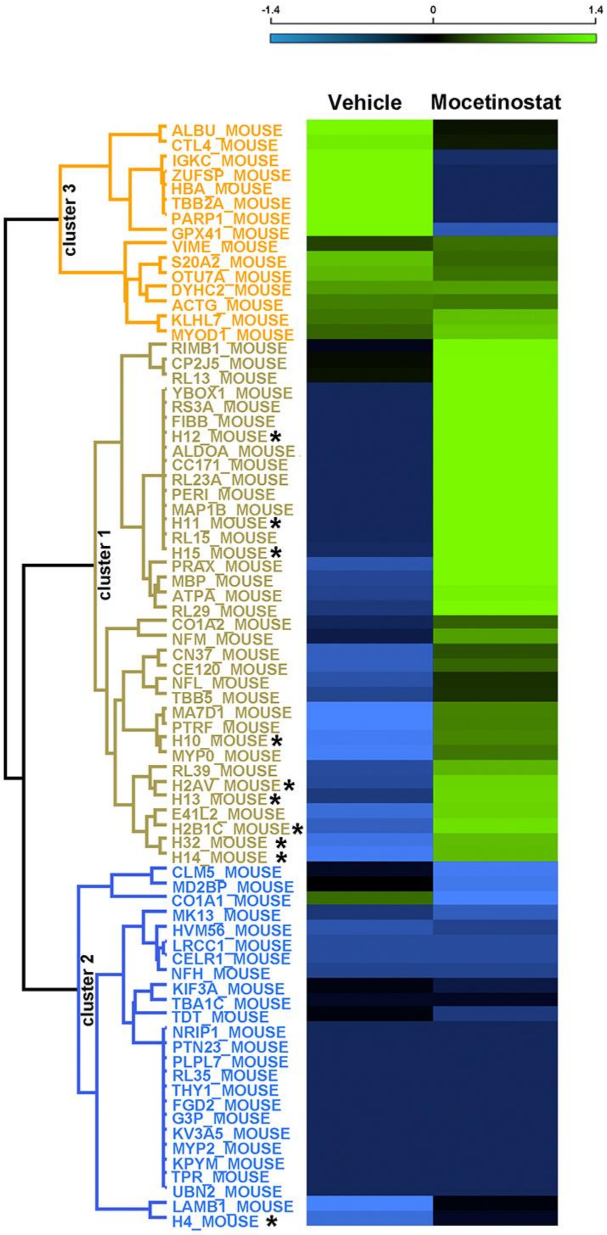

Supplementary Fig. 15 Clustering of protein abundances in adult mouse sciatic nerves after Mocetinostat treatment or its vehicle for 1 day post lesion. Heat map representing label-free protein quantities as determined by MaxQuant LFQ algorithm from mass spectrometry analysis of immunoprecipitates with an anti-acetylated lysine antibody. Sciatic nerves of mice treated for 1 day after lesion with Mocetinostat or its vehicle were lysed and used for immunoprecipitation. Proteins could be grouped in 3 different clusters according to intensity patterns, with cluster 1 containing proteins with increased abundance after Mocetinostat treatment compared to Vehicle. According to UniprotKB protein annotations, 31% of proteins in cluster 1 have known lysine acetylation sites (including several histones*), while only 16% and 13% in clusters 2 and 3, respectively. This result suggests efficient HDAC1/2 inhibition by Mocetinostat treatment.

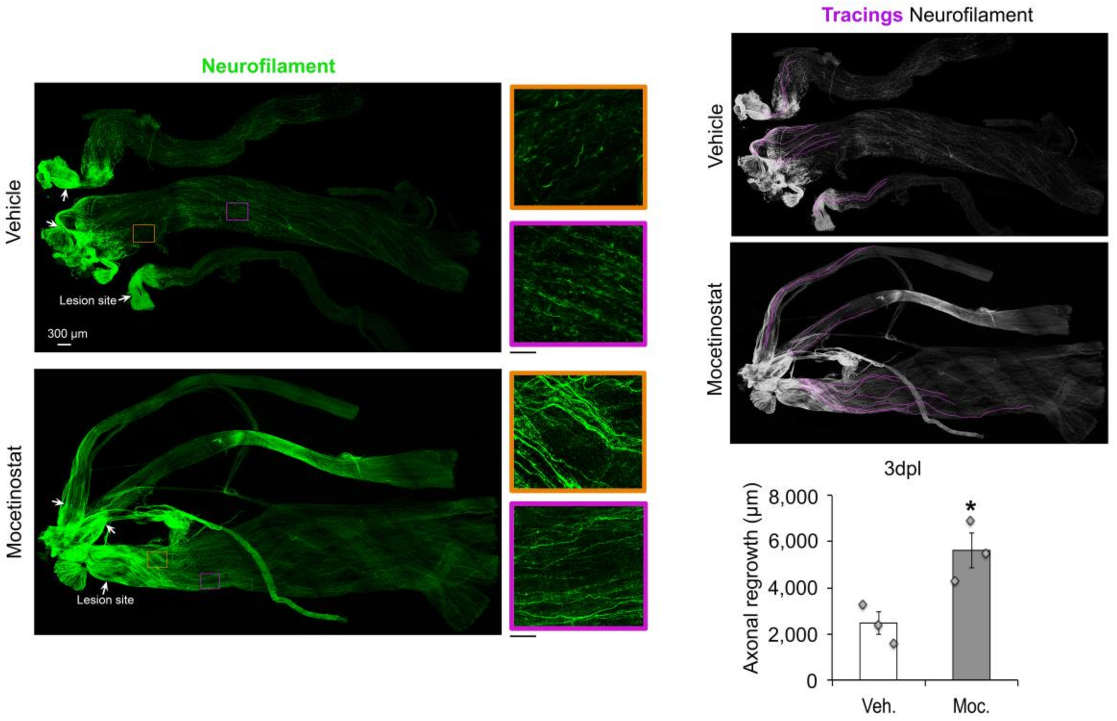

Supplementary Fig. 16 HDAC1/2 inhibitor treatment accelerates axonal regrowth after lesion. Z-series projections (confocal stacks) of whole-nerve Neurofilament immunofluorescence in nerves of vehicle and Mocetinostat-treated mice and axonal tracings (acquired using NeuronJ) quantifying axonal regrowth from the lesion site (white arrows). Scale bars of colored boxes=100 µm. Unpaired two-tailed Student's *t*-tests, *P* value=0.02. Values=mean, error bars=s.e.m. Sample size: n=3 animals per group; number of tracings: 10 to 11 per nerve.

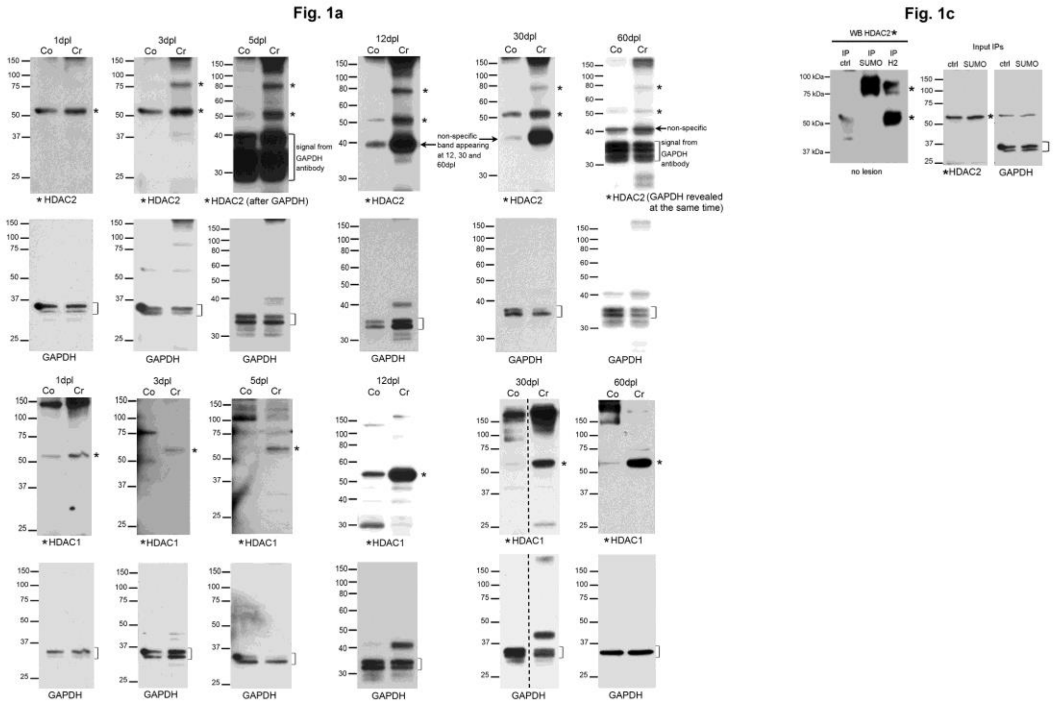

Supplementary Fig. 17 Full-size blots corresponding to cropped images presented in Fig. 1. Western blot images. Asterisks or brackets indicate specific band(s) recognized by the antibody, dashed lines indicate that samples have been run on the same gel, but not on consecutive lanes.

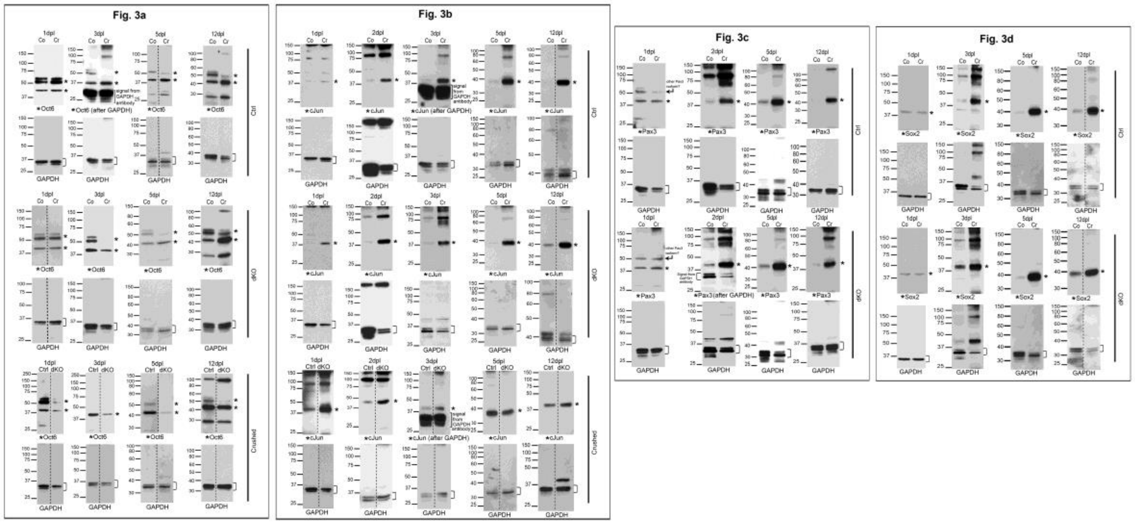

Supplementary Fig. 18 Full-size blots corresponding to cropped images presented in Fig. 3. Western blot images. Asterisks or brackets indicate specific band(s) recognized by the antibody, dashed lines indicate that samples have been run on the same gel, but not on consecutive lanes.

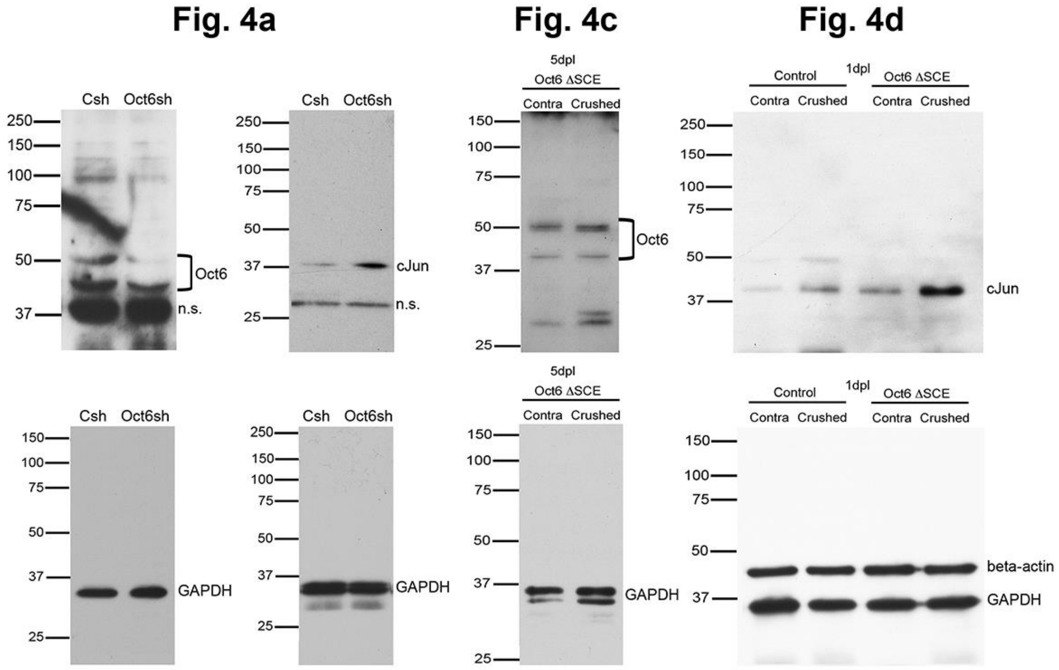

Supplementary Fig. 19 Full-size blots corresponding to cropped images presented in Fig. 4. Western blot images. Position of protein name or brackets indicate specific band(s) recognized by the antibody, n.s. = non specific band.

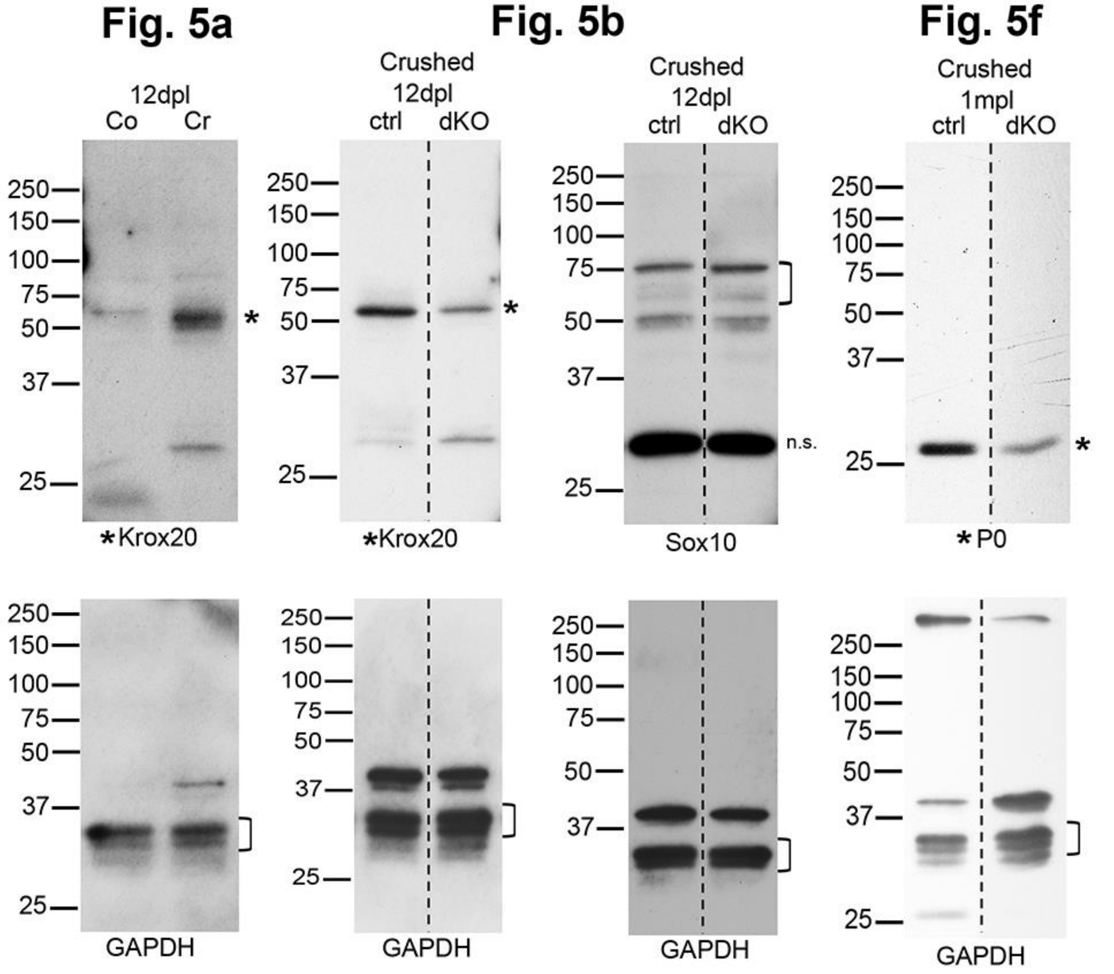

Supplementary Fig. 20 Full-size blots corresponding to cropped images presented in Fig. 5. Western blot images. Asterisks or brackets indicate specific band(s) recognized by the antibody, n.s. = non specific band.

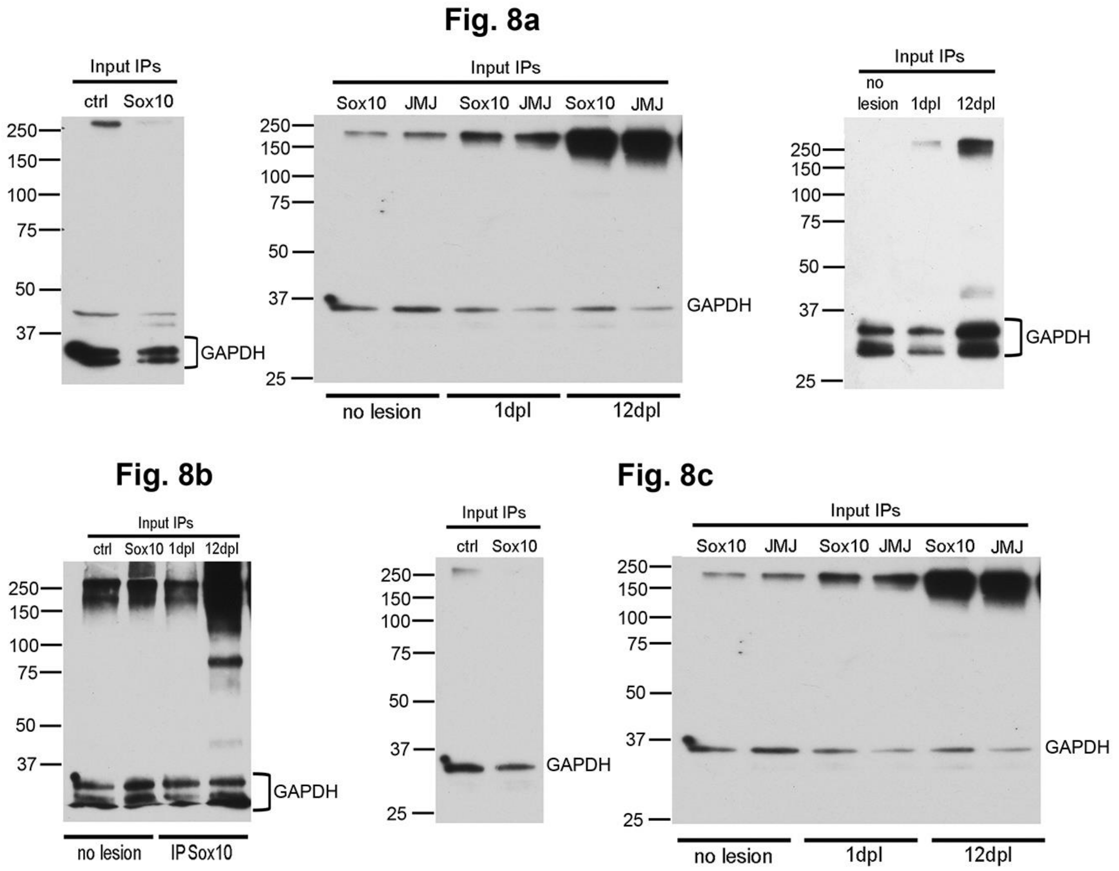

Supplementary Fig. 21 Full-size blots corresponding to cropped images presented in Fig. 8. Western blot images. Position of protein name or brackets indicate specific band(s) recognized by the antibody.

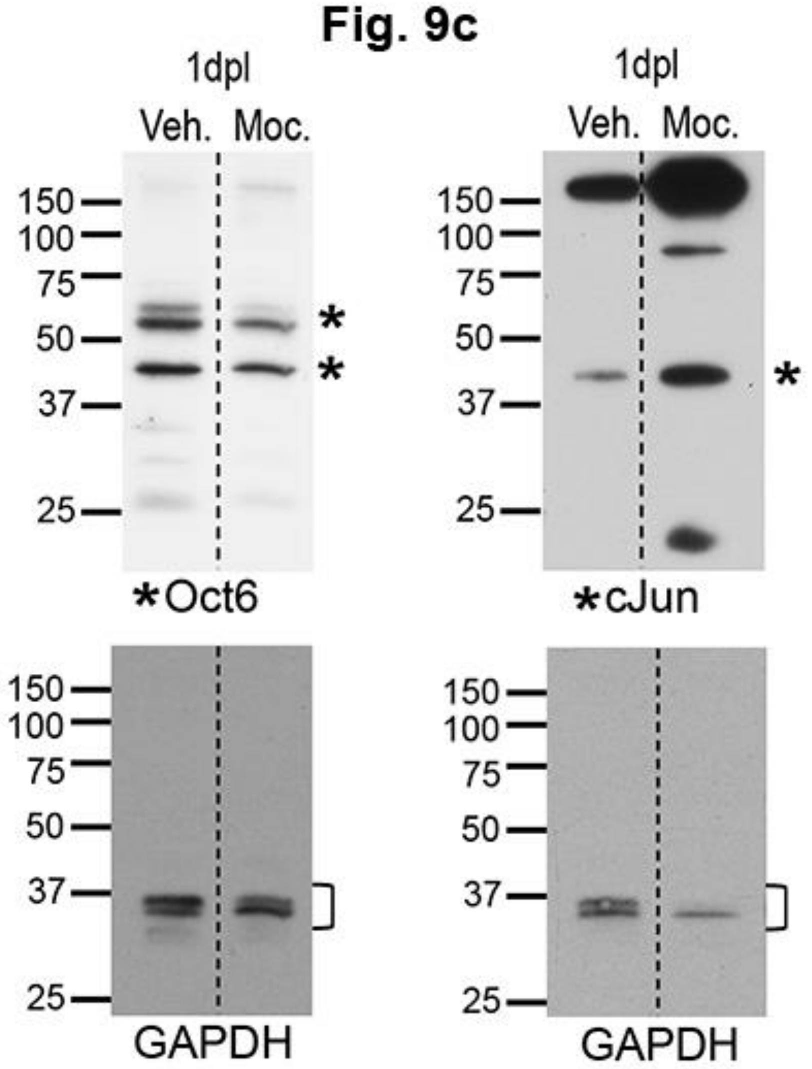

Supplementary Fig. 22 Full-size blots corresponding to cropped images presented in Fig. 9. Western blot images. Asterisks or brackets indicate specific band(s) recognized by the antibody.

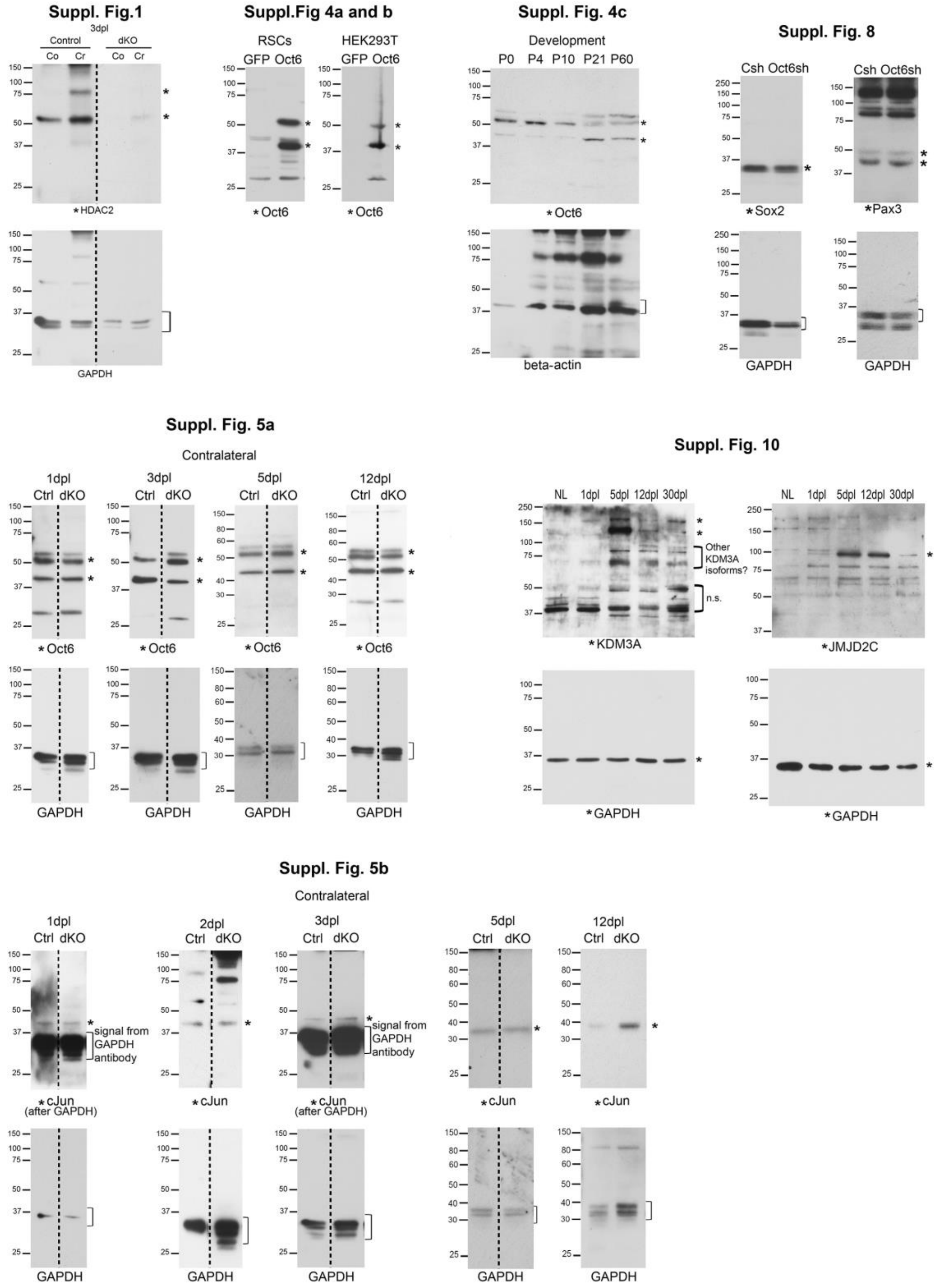

Supplementary Fig. 23 Full-size blots corresponding to cropped images presented in supplementary Figs. 1-4-5-8-10. Western blot images. Asterisks or brackets indicate specific band(s) recognized by the antibody, n.s. = non-specific band.
